# Longitudinal study of the manifestations and mechanisms of technology-related prescribing errors in pediatrics

**DOI:** 10.1093/jamia/ocae218

**Published:** 2024-09-11

**Authors:** Magdalena Z Raban, Erin Fitzpatrick, Alison Merchant, Bayzidur Rahman, Tim Badgery-Parker, Ling Li, Melissa T Baysari, Peter Barclay, Michael Dickinson, Virginia Mumford, Johanna I Westbrook

**Affiliations:** Centre for Health Systems and Safety Research, Australian Institute of Health Innovation, Macquarie University, Sydney, New South Wales 2109, Australia; Centre for Health Systems and Safety Research, Australian Institute of Health Innovation, Macquarie University, Sydney, New South Wales 2109, Australia; Centre for Health Systems and Safety Research, Australian Institute of Health Innovation, Macquarie University, Sydney, New South Wales 2109, Australia; Centre for Health Systems and Safety Research, Australian Institute of Health Innovation, Macquarie University, Sydney, New South Wales 2109, Australia; Centre for Health Systems and Safety Research, Australian Institute of Health Innovation, Macquarie University, Sydney, New South Wales 2109, Australia; Centre for Health Systems and Safety Research, Australian Institute of Health Innovation, Macquarie University, Sydney, New South Wales 2109, Australia; School of Medical Sciences, Biomedical Informatics and Digital Health, Faculty of Medicine and Health, The University of Sydney, New South Wales 2006, Australia; Department of Pharmacy, The Sydney Children’s Hospital Network, Sydney, New South Wales 2145, Australia; Digital Health Services, South Western Sydney Local Health District, Sydney, New South Wales 2170, Australia; Centre for Health Systems and Safety Research, Australian Institute of Health Innovation, Macquarie University, Sydney, New South Wales 2109, Australia; Centre for Health Systems and Safety Research, Australian Institute of Health Innovation, Macquarie University, Sydney, New South Wales 2109, Australia

**Keywords:** electronic health records, medication errors, informatics, patient safety, user-centered design

## Abstract

**Objectives:**

To examine changes in technology-related errors (TREs), their manifestations and underlying mechanisms at 3 time points after the implementation of computerized provider order entry (CPOE) in an electronic health record; and evaluate the clinical decision support (CDS) available to mitigate the TREs at 5-years post-CPOE.

**Materials and Methods:**

Prescribing errors (*n* = 1315) of moderate, major, or serious potential harm identified through review of 35 322 orders at 3 time points (immediately, 1-year, and 4-years post-CPOE) were assessed to identify TREs at a tertiary pediatric hospital. TREs were coded using the Technology-Related Error Mechanism classification. TRE rates, percentage of prescribing errors that were TREs, and mechanism rates were compared over time. Each TRE was tested in the CPOE 5-years post-implementation to assess the availability of CDS to mitigate the error.

**Results:**

TREs accounted for 32.5% (*n* = 428) of prescribing errors; an adjusted rate of 1.49 TREs/100 orders (95% confidence interval [CI]: 1.06, 1.92). At 1-year post-CPOE, the rate of TREs was 40% lower than immediately post (incident rate ratio [IRR]: 0.60; 95% CI: 0.41, 0.89). However, at 4-years post, the TRE rate was not significantly different to baseline (IRR: 0.80; 95% CI: 0.59, 1.08). “New workflows required by the CPOE” was the most frequent TRE mechanism at all time points. CDS was available to mitigate 32.7% of TREs.

**Discussion:**

In a pediatric setting, TREs persisted 4-years post-CPOE with no difference in the rate compared to immediately post-CPOE.

**Conclusion:**

Greater attention is required to address TREs to enhance the safety benefits of systems.

## Introduction

While studies have shown that some prescribing errors may be addressed following the implementation of computerized provider order entry (CPOE) in electronic health records in hospitals,^1–5^ new technology-related medication error types are also well documented in the literature.^6–13^ These technology-related errors (TREs) arise from the design, functionality, or use of the CPOE system and the embedded clinical decision support (CDS). A systematic review of 14 studies estimated that TREs account for a median of 26.1% (interquartile range: 17.6-42.1) of all prescribing errors in hospitals with a CPOE, but have been reported to be as high as 77%.^6^ A further review of technology-related medication errors (including those in medication administration and prescribing) highlighted that the majority of research on TREs has been conducted in the first 2 years post-CPOE implementation, but that TREs have also been shown to persist even beyond 10 years post-CPOE.^14^ There can be a period of adjusting to a new system immediately post-CPOE implementation or after CPOE upgrades,^15^ and some studies have shown prescribing errors increase in the early weeks post-implementation.^3^ Prescribing errors in the early periods of CPOE use that are technology-related may be expected to decline as users become more familiar with system functionality. However, underlying error mechanisms, such as incorrect selection of an option from a drop-down menu, have been described by multiple studies irrespective of how long the CPOE has been in use.^14^ A clear understanding of how TREs change over time is lacking as no study has examined TREs longitudinally at the same site. Such information is important in determining how to systematically address TREs through CPOE and CDS modifications and better design.^14^ Addressing TREs could significantly optimize safety benefits of CPOE systems.

A further gap in the evidence-base is the lack of studies investigating TREs in pediatric settings. Pediatric prescribing presents specific challenges, such as weight-based doses that can change due to age-related physiological changes at different stages of development. Thus, CPOE systems for pediatric settings need to incorporate specific functions and CDS to support pediatric prescribing (eg, dose calculators, dose rounding rules), and hence differ from CPOE systems designed for adult populations.^16^^,^^17^ Only a small number of studies have examined TREs in pediatrics. The largest study was from the United States, published in 2006, and reviewed 6916 medication orders for 352 pediatric inpatients.^18^ The study reported that 19% of prescribing errors 12-months post-CPOE were technology-related, with incorrect selection from a drop-down menu the most frequent error mechanism, followed by errors arising from the use of order sets.^18^ Three other studies examined incident reports and reported issues such as rounding rules for dose calculators resulting in dose errors.^19^ Two studies of incident reports found that 36%^17^ and 45% of incidents were CPOE-related.^20^ While incident reports can be informative, it is well established that they vastly underestimate rates of medication errors due to underreporting, and thus may not provide a complete picture of the types of TREs occurring.^21^ Thus, there is limited evidence on the rates of TREs in the pediatric setting, including how these change over time. This is despite the more complex CDS and functionality requirements for CPOE in the pediatric setting, and the potentially serious impact of medication errors for children in hospital.^19^^,^^22^

Our aims were to describe the TREs occurring during medication prescribing, including their manifestations and mechanisms (how they occurred) at 3 time points—immediately, 1-year, and 4-years post-CPOE implementation—at a large tertiary pediatric hospital, to assess changes over time. As CPOE systems, and their embedded CDS, are continually evolving, periodic testing of systems to understand whether the CDS is addressing errors has been shown to be a valuable tool that can lead to improvements in prevention of errors.^23^^,^^24^ Thus, we also examined whether the CPOE system 5-years post-implementation had CDS to mitigate against TREs identified in the previous study periods.

## Methods

### Study design

We used retrospective chart review at 3 time points to identify clinical prescribing errors that were technology-related. The CPOE testing environment of the live system at 5-years post-CPOE implementation was used to assess the CDS available to mitigate against TREs. We used a dual-classification system for TREs that codes the manifestation (clinical error type) and mechanism (how the error occurred) for each TRE.^7^^,^^25^

### Setting and CPOE system

The study was conducted on 9 general medical and surgical wards of a 300-bed tertiary pediatric hospital in Australia.

During 2016, the hospital added electronic medication management to allow electronic prescribing and medication administration, to a pre-existing electronic medical record (Cerner Millenium). Almost all medication prescribing was transitioned from paper to the CPOE, including intravenous fluids and the majority of complex medications, including titratable infusions. Paper prescribing for complex opioid infusions, insulin pumps, and insulin sliding scale orders remained in place during the entire study period. The CDS in the CPOE included order sentences; pre-built complex titratable infusion orders; a dose calculator; age and weight filtering of order sentences; order sets for pre- and post-surgical admissions; asthma management; day-stay infusions and nurse-initiated medications; and allergy and drug-drug interaction checking (set at the Multum database “severe-contraindicated” level).

### Prescribing error data

Data on prescribing errors that occurred over 3 time points were obtained from a study which aimed to assess the impact of the introduction of the CPOE on medication errors at the hospital.^3^^,^^26^ The 3 time points were: (1) immediately post-CPOE (first 70 days post-implementation in May-July 2016 using data from a stepped-wedge cluster randomized control trial); (2) 1-year post (June-September 2017), and (3) 4-years post (June-September 2020).^3^ During the first study period, all patient admissions on the study wards were included. During the 1-year and 4-year post-implementation study periods, we randomly selected patient admissions from each study ward to ensure the sample represented the distribution of study wards across the hospital.

In each study period, clinical research pharmacists reviewed all medication orders for the included admissions to identify and classify prescribing errors (eg, wrong dose, wrong drug) (Supplementary file 1). For all errors, potential harm was rated on a 5-point scale: 1-minimum potential harm; 2-minor; 3-moderate; 4-major; and 5-serious (definitions in Supplementary file 1). Prescribing errors were identified for 2263 admissions during the stepped-wedge cluster randomized trial period (immediately post-CPOE), 1189 admissions at 1-year post-CPOE, and 1143 admissions at 4-years post-CPOE. In total, 35 322 medication orders were reviewed to identify prescribing errors post-CPOE. For the current study, we extracted information about all the clinical prescribing errors with a potential harm rating of moderate, major, or serious (rating of 3+, *n* = 1315). This dataset comprised clinical prescribing errors in the CPOE immediately (*n* = 744), 1-year (*n* = 299), and 4-years (*n* = 272) post-CPOE implementation. Technology-related errors were only assessed for those orders placed in the CPOE and orders on paper charts (eg, due to system downtime) were excluded.

### Identification of TREs and their underlying mechanisms

Two clinical pharmacists independently assessed prescribing errors to determine whether each error was technology-related. TREs were defined as errors where there was a high probability that the functionality or design of the CPOE contributed to the error.^7^ TREs included errors arising from new work processes and changes in prescribing practices required when using the CPOE.^7^ To identify TREs, clinical research pharmacists had access to details of the medication orders with errors, as well as the patient’s medical record. Pharmacist reviewers also had access to the CPOE testing environment allowing errors to be replicated in order to identify the CPOE features that may have contributed to the TRE. Further details of this process are reported elsewhere.^25^

Once an error was identified as a TRE, the Technology-Related Error Mechanism (TREM) classification was applied to code the underlying error mechanisms.^25^ TREM includes 7 major mechanism categories (Table 1) with 19 subcategories (details in Supplementary file 2). Multiple mechanisms could be assigned to a single error. Inter-rater reliability assessment for TRE identification (kappa 0.79, 95% confidence interval [CI]: 0.71, 0.88) and mechanism classification (kappa 0.80, 95% CI: 0.68, 0.92) showed moderate to strong reliability.

**Table 1.** Major categories of the technology-related error mechanism classification.^25^^a^

Mechanism category	Definition
1. Incorrect system configuration or system malfunction	Errors within the system itself, such as errors in pre-programmed order sentences.
2. Prescribing on the wrong patient record	Errors that occur when prescriber uses an incorrect patient record and prescribes medication clearly not intended for the patient.
3. Selection errors	Errors that occur when any element during prescribing is selected incorrectly from pre-programmed options presented by the system, eg, from a drop-down menu.
4. Construction errors	Errors that occur when constructing an order or typing free text, rather than selecting from drop-down lists or editing order sentences.
5. Editing errors	Errors that occur when editing (or not editing) a selected prepopulated order sentence or existing order (that are not selection errors or construction errors) eg, ondansetron intravenous order sentence is selected and then was edited to oral without removing “infuse over 15 minutes” from the order comments.
6. Errors that occur when using workflows that differ from a paper-based system	Examples include inadvertently creating inpatient orders when attempting to order discharge medication, and failure to refresh the medication profile resulting in missing recent medication changes and thus, prescribing in error.
7. Use of hybrid systems (contributing factor)	Errors that occur when 2 different systems are used for prescribing including some prescribing remaining on paper medication charts or the use of different electronic systems.

a Full classification shown in Supplementary files.

### Assessment of CDS to mitigate TREs

To assess whether the CPOE system 5-years post-implementation (2021) had CDS to mitigate against TREs identified in the previous study periods, clinical research pharmacists entered each medication order with a TRE into the CPOE testing environment to determine if any CDS was provided to the user to guard against the TRE.

This assessment was based on the main mechanism of the TRE. For example, if a TRE was due to incorrect selection from a drop-down menu, the absence of the incorrect option in the drop-down menu was identified as decision support that could mitigate the error. When an order had multiple TREs, the level of decision support available for each of the clinical errors was assessed separately. If multiple TRE mechanisms had been assigned to an error, the decision support that would guard against the first of these in the sequence was recorded. That is, only one level of decision support was assigned for each TRE, irrespective of the number of mechanisms associated with the error. The type of CDS was classified based on an existing classification: none, guided, information alert, permitted alert, or restricted (Table 2).^27^

**Table 2.** Definitions of levels of clinical decision support adapted from Pontefract et al^27^

Level of decision support	Description	Examples of decision support in this study
Restricted	The prescriber is prevented from proceeding with the prescription with a hard-stop alert; or there is no option to prescribe a particular route, frequency or dose.	Route option/s for specific medications unavailable Not allowing an order to be signed without calculating weight-based dose
Permitted alert (with reason for override)	An alert appears and a reason needs to be given by the prescriber to override the alert and progress.	Drug-drug interaction and allergy alerts that require selection of a reason from a drop-down list
Information alert	An alert appears, but the prescriber does not need to add a reason to override the alert and proceed.	Pop-up alert when prescribing sodium chloride 3% infusion to inform prescriber of restriction to use within intensive care Alert to refresh record after modifying medication chart
Guided	Elements of the prescription order are auto-populated for the prescriber (eg, dose, frequency, route), or highlighting techniques are used to guide the user, eg, through use of different font.	Indication specific order sentences Use of red font to distinguish specific medications in drop-down menus eg, high alert clonazepam
None	No CDS appears at the point of prescribing.	

### Statistical analyses

The TRE rate and percentage of clinical prescribing errors that were technology-related for each period were calculated. The TRE rate was defined as the number of TREs per 100 orders and was calculated overall for all TRE orders and by mechanism categories. We estimated the crude TRE rate/100 orders and their 95% CI by fitting a simple Poisson model. The outcome variable was the number of TREs per patient-day, with an offset of the logarithm of the number of orders per patient-day.

Our data has a 3-level hierarchical structure: the lowest level is daily TRE counts and total orders for each patient. Thus, TRE counts are clustered within patients and patients are clustered within wards. To examine how the TRE rate changed over time, we estimated the incidence rate ratio of TREs for each study period (with immediately post-CPOE as the reference) by fitting a 3-level random intercept multivariable Poisson model with random intercepts at 2 nested levels (ward and patient). To adjust for patients’ demographic characteristics that might have affected the TRE rates we also included sex and age with restricted cubic spline using 4 knots in the model. Cubic spline of age was included in the model to account for the non-linear relationship between age and TRE rate.^28^ The outcome variable was highly over dispersed with the dispersion parameter *α* = 7.5 as opposed to zero (95% CI: 5.6, 10.0). This was handled by estimating robust standard error with “vce(robust)” option in “mepoisson” command in Stata (21). As of the current state of development in statistics, there is no option to estimate adjusted rates from a multilevel Poisson model with random intercept to adjust the standard error for clustering. For that reason, we adopted a separate model to estimate the adjusted TRE rates and their 95% CI for each period. A multivariable Poisson model, with the same outcome and offset variables as in the previous model, was fitted including study period, sex, and restricted cubic spline of age of the participant as explanatory variables. To adjust the standard error of the predicted rate for clustering we used patient ID and ward ID as the clustering variables. A community contributed prefix command “vcemway” in Stata was adopted to adjust for multi-way clustering by patient ID and ward.^29^ Data manipulation was done in SAS 9.4 and analyses were conducted using Stata version 18.

## Results

The sample characteristics are shown in Supplementary file 3.

### TRE prescribing error rates

The overall adjusted TRE rate was 1.23 TREs/100 orders (95% CI: 1.01, 1.44). The TRE rate decreased from 1.49 TREs/100 orders immediately post-CPOE to 0.87 TREs/100 orders 1-year post-CPOE (incident rate ratio [IRR]: 0.60; 95% CI: 0.41, 0.89; Table 3). At 4-years post-CPOE the TRE rate was not significantly different from the rate immediately post-CPOE (IRR: 0.80; 95% CI: 0.59, 1.08).

**Table 3.** Technology-related error rates immediately, 1-year, and 4-years post-CPOE implementation and adjusted incidence rate ratios comparing study periods.

Study period	Number of orders reviewed	Number of errors that were TREs	Number of TREs/100 orders (95% CI)	**Adjusted** ^a^ **TRE rates (95% CI)**	Adjusted IRR (95% CI)
Immediately post	15 844	233	1.47 (1.29, 1.67)	1.49 (1.06, 1.92)	1.00
1-year post	8882	77	0.87 (0.68, 1.08)	0.87 (0.61, 1.14)	0.60 (0.41, 0.89)
4 years post	10 596	118	1.11 (0.92, 1.33)	1.11 (0.86, 1.37)	0.80 (0.59, 1.08)
**Total**	**35 322**	**428**	**1.21 (1.10, 1.33)**	**1.22 (1.00, 1.44)**	

a Number of TREs/100 orders after adjusting for patient age, sex, ward, and patient level clustering.

Abbreviations: CI = confidence interval; CPOE = computerized provider order entry; IRR = incidence rate ratio; TRE = technology-related error.

TREs accounted for 32.9% of all prescribing errors in our sample over all study periods. Table 4 shows the percentage of errors that were TREs for each study period by the potential harm rating of the errors. At 4-years post-CPOE, TREs made up the greatest percentage of all prescribing errors (43.4%) compared to all other study periods (31.3% immediately post- and 25.8% at 1-year post; Table 4). Across all study periods, few TREs were rated as having potentially serious harm, defined as an error which has the potential to result in a patient’s death.

**Table 4.** Percentage of clinical prescribing errors that were technology-related immediately, 1-year post, and 4-years post-CPOE by potential harm rating.

Potential harm rating of error	Immediately post			1-year post			4-years post		
	No. of prescribing errors	No. of TREs	% of errors that were TREs	No. of errors	No. of TREs	% of errors that were TREs	No. of errors	No. of TREs	% of errors that were TREs
Moderate	620	151	24.4	266	62	23.3	244	96	39.3
Major	110	69	62.7	28	12	42.9	26	21	80.8
Serious	14	13	92.9	5	3	60.0	2	1	50.0
**Total**	**744**	**233**	**31.3**	**299**	**77**	**25.8**	**272**	**118**	**43.4**

Abbreviations: CI = confidence interval; CPOE = computerized provider order entry; TRE = technology-related error.

### Types of prescribing errors and medications associated with TREs

**Figure 1.**
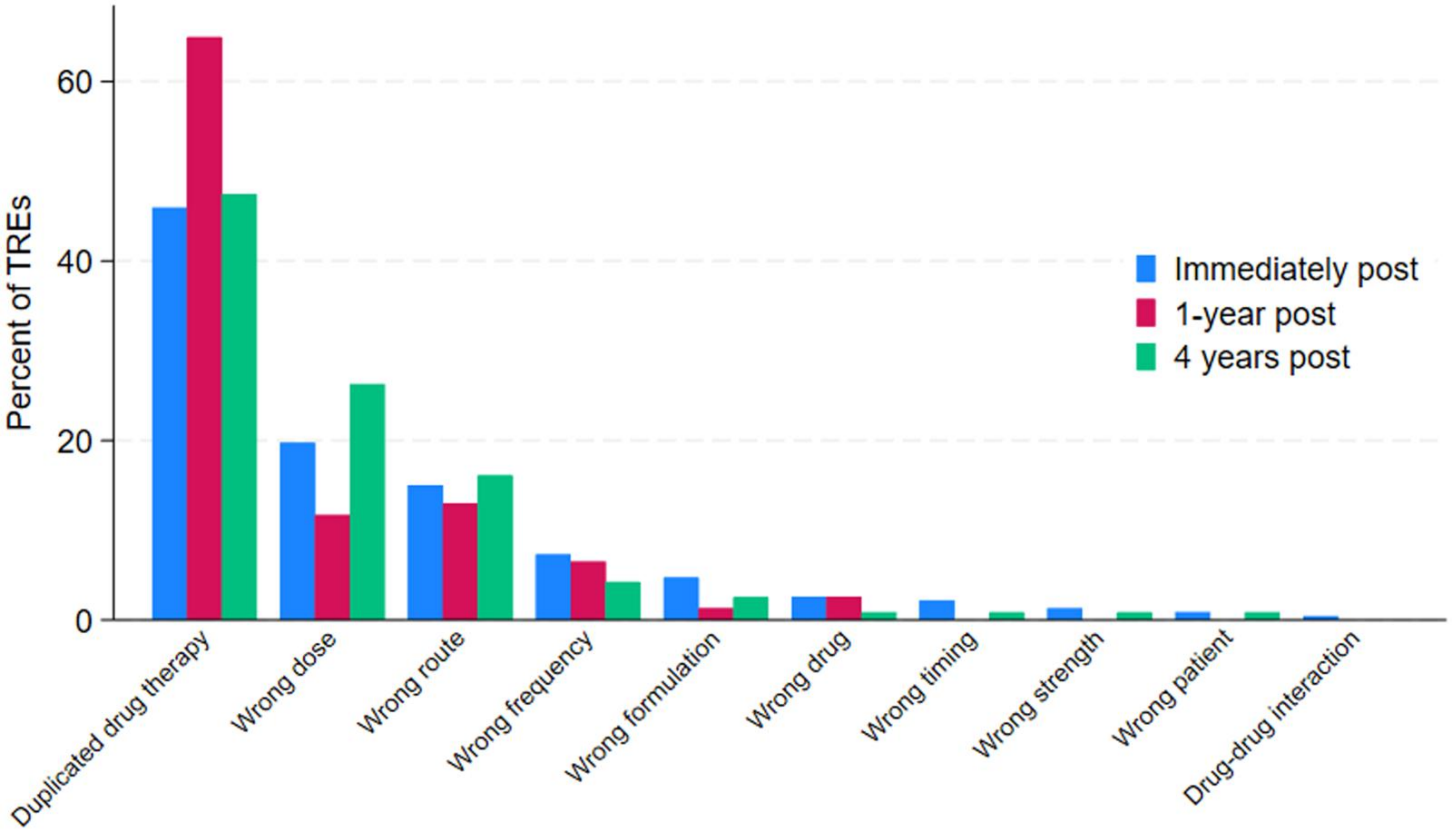
Distribution of technology-related errors by their manifestation (ie, clinical error type) immediately, 1-year, and 4-years post-CPOE (*n* = 428).

Figure 1 shows the distribution of TREs by their manifestation, that is, the clinical error type. Duplicated drug therapy errors were the most frequent technology-related clinical error type across all study periods and particularly at 1-year post-, followed by wrong dose and wrong route. TRE rates by clinical error category are shown in Supplementary file 4. Despite TREs resulting in duplicate drug therapy errors making up a higher proportion at 1-year post-CPOE compared to other study periods (Figure 1), the rate of duplicate drug therapy errors did not change over time (Supplementary file 3). Notably, the rate of TREs that were wrong dose errors changed over the study periods, with a decrease at 1-year post-CPOE compared with immediately post-CPOE, and then an increase at 4-years post-CPOE.

The most frequent medication with a TRE was oxycodone across all study periods (Supplementary file 5). Among the 5 most frequent medications with TREs across all study periods, the majority were high-risk medicines, including insulin, fentanyl, vancomycin, paracetamol, and midazolam.

### The underlying mechanisms of TREs

**Figure 2.**
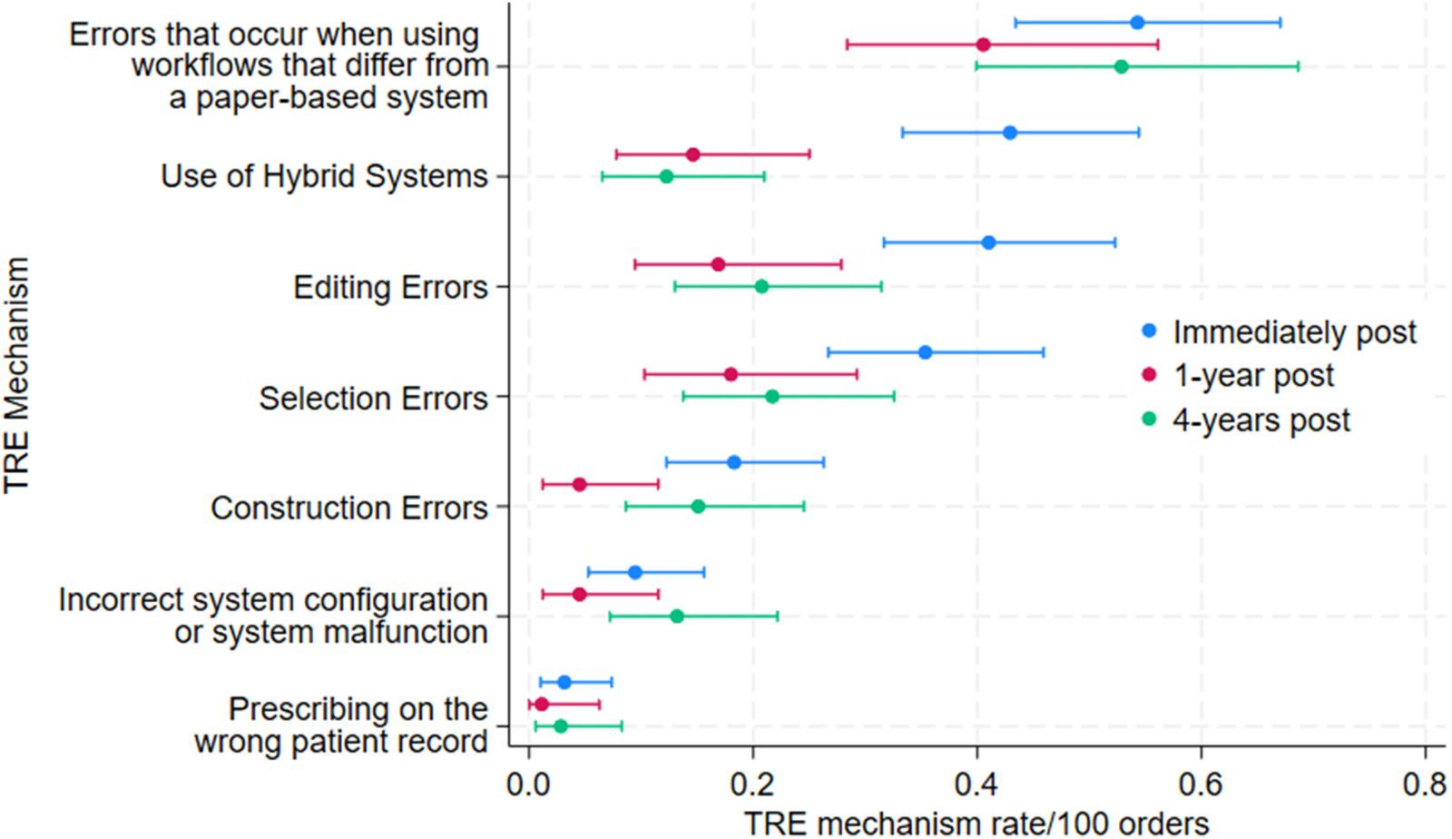
Technology-related error (TRE) mechanism rates by study period.

Figure 2 shows the TRE mechanism rates (details are presented in Supplementary file 6). Of the total 428 TREs identified, 120 TREs (28.0%) were assigned more than one mechanism across all study periods. The most frequent TRE mechanism in each study period was “errors that occur when using workflows that differ from a paper-based system”. The rate for this mechanism did not change significantly across the study periods (Figure 2). Within this mechanism category, “updated medication profile, active workspace or medication chart not viewed prior to ordering” was the most frequent sub-category and often occurred when prescribing via an order set which limited the clinicians view of the active medication chart.

Immediately post-CPOE, the “use of hybrid systems” mechanism had the second highest rate (0.43/100 orders; 95% CI: 0.33, 0.54). Within this category, the predominant sub-category was “errors occurring during initial system roll-out”, which occurred when orders were transcribed from paper charts to the CPOE for the first time. The mechanism sub-category “errors occurring when paper charts are used for some prescribing” was also frequent in the “hybrid systems” mechanism category. This included errors that occurred when paper charts were used for specialized prescribing (eg, pain charts). However, “hybrid systems” as a contributor to TREs significantly decreased from period 1 to 2 and remained at this lower rate in period 3 (Figure 2).

“Editing errors” was a mechanism category that had a higher rate immediately post-CPOE compared to 1-year, with a non-significant increase from 1-year post- to 4-years post-CPOE. These errors occurred when prescribers attempted to edit order details, edited within a dose calculator, edited to correct a previous TRE, attempted to edit a default time or date, and when there was misuse of order actions on existing orders (eg, inadvertently reordering a medication instead of discontinuing it). “Selection errors” showed a similar pattern to editing errors, with a higher rate immediately post-CPOE compared with 1-year post-CPOE, and a slight non-significant increase from 1-year post- to 4-years post-CPOE. This mechanism included incorrect selection from drop-down menus, for example, selecting the wrong order sentence from the options displayed.

### CDS available to mitigate TREs

Entering each of the 428 medication orders with TREs into the CPOE system in 2021, 5-years post-implementation, showed that 32.5% (*n* = 140) should have been prevented if the current CDS in the system was used as intended. Guided decision support (eg, order sentences) was available for 99 TREs (23.0%); an information alert for 30 TREs (7.0%); restricted decision support (ie, hard-stops in the CPOE) for 10 TREs (2.3%); and a permitted alert (requiring a reason to be entered when overriding the alert) for 1 TRE (0.2%). Supplementary file 7a shows details.

An example of restricted decision support was functionality that prevented prescribers from leaving the final dose in the order as mg/kg instead of the total calculated dose. Guided decision support predominantly involved the use of order sentences. Examples of information alerts included 2 alerts to ensure a correct dose is entered for a medication order including an alert when a prescriber attempted to construct an order with a free-text dose or dosing unit, and an alert when a dose with more than 2 decimal places was entered.

The TRE mechanisms where limited CDS was included related to “not viewing the updated medication profile prior to ordering”, “errors when using tasks and reminders”, and “selection errors when ordering” (Supplementary file 7b).

## Discussion

We found that at least 1 in 3 prescribing errors with a potential for moderate, major, or serious patient harm were technology-related at a tertiary pediatric hospital. Importantly, the TRE rate was highest immediately post-CPOE and the risk of TREs decreased by 40% at 1-year post-CPOE. However, the overall rate increased at 4-years post-CPOE to a rate similar to that immediately post-CPOE. These results confirm that TREs persist for many years post-implementation. Our assessment of the extent to which decision support in the system 5-years post-CPOE may prevent previously identified TREs found that only a third may have been prevented.

Comparison of the rates of TREs among studies is difficult due to the use of varying methods and definitions to detect and report TREs. The most comparable study of TREs was conducted at 2 adult hospitals with 2 different CPOE systems. That study found that TREs comprised 42% of prescribing errors of all severities (78 per 100 inpatient admissions); however, only 2.2% were rated as potentially serious.^7^ This is in contrast to our results, where we only examined potentially serious errors and found up to 40% to be technology-related. There are also differences in the most frequent TRE mechanisms in our study compared with the results from the 2 adult hospitals where selection errors were the most frequent mechanism.^7^ Our results show that errors due to new workflows were the most frequent mechanism. These differences among studies highlight that TREs vary between settings, in this case the pediatric and adult setting, and the need for site specific monitoring of technology-related issues to improve medication and patient safety.

Our results showed that while there was a reduction in TRE rates 1-year post-CPOE implementation, the rate rose again at 4-years post-implementation and was not significantly different to the rate immediately post-CPOE. Furthermore, the profile of the predominant underlying mechanisms of the TREs showed minimal change over time, with errors due to workflow changes, errors due to hybrid systems, editing errors and selection errors the mechanism categories with the highest rates across all study periods. A number of factors may have contributed to this longer-term stagnation of TRE rates. Firstly, while CPOE systems undergo ongoing upgrades and optimization, the TREs and their mechanisms may have not been readily identifiable to CPOE system managers. Research suggests that CPOE optimization occurs based on a variety of sources such as user requests, organization projects, and incident reports.^30^ While all these sources offer valuable insights into CPOE usability issues, they cannot estimate the relative frequency of the CPOE issues as they are not representative data sources on medication errors. This limits the ability to prioritize optimization activities, which is a key challenge for health information technology (HIT) managers.^30^ For example, TREs with a potentially high risk for patient harm should be prioritized, however, in addition, the TRE mechanisms that are frequent should also be targeted as these may be contributing to poor system usability and frequent errors. Secondly, hospitals undergo staff changes and turnover, including new junior doctors joining teams on an annual basis. New staff undergo CPOE training; however, our results highlight specific areas that may require greater attention in training. For example, in each of the study periods we found TREs occurred when not viewing the updated medication profile. Staff onboarding activities could include guidance for prescribers on how to avoid these errors. Crucially, however, TREs should be resolved through CPOE changes as this is a more sustainable solution than continual staff training. Nonetheless, training staff may be necessary when changes to the CPOE are not possible at the local level.

We found that TREs were most likely to result in duplicate drug therapy errors. Other studies have also reported a substantial increase in duplicate drug therapy errors following CPOE.^31–35^ Without understanding the underlying mechanisms of these TREs, the implementation of duplicate alerts in the CPOE may appear to be viable solution. However, turning duplicate drug alerts on in CPOE is known to generate a large volume of clinically irrelevant, interruptive alerts which contribute to clinician dissatisfaction and alert fatigue.^36^^,^^37^ Examining the underlying mechanisms of these errors provides other avenues for strategies to address these errors. For example, some of the duplicate drug therapy errors in our study were due to the use of hybrid systems where some prescribing remained on paper charts. The implementation of a duplicate alert would not address these errors as the CPOE would not include what is prescribed on a paper chart. Another example is duplicate drug therapy errors that occur when 2 prescribers may be accessing a patient’s chart at the same time. If a prescriber has a chart open, but fails to refresh their view of current orders prior to signing off on changes, they will not see recent medication changes made by another prescriber. Similar causes and solutions with duplicate errors have been highlighted by other studies.^33^^,^^34^

The available CDS in the CPOE at the time of assessment addressed 32.5% of the TREs. Examples of the CDS provide some guidance on what may be effective in reducing TREs. It was also positive to see that the majority of this CDS was in the “guided” decision support category, that is, did not use interruptive alerts. However, information alerts, that is, one type of interruptive alert, were the second most frequent form of CDS in the CPOE to address the TREs. It is well known that interruptive alerts are not favored by clinicians as a form of CDS and that there is overuse of interruptive alerts, leading to alert fatigue and habitual overriding behavior.^36^^,^^38^ Thus, strategies to mitigate TREs should avoid interruptive alerts where possible.^38^

The large sample of prescribing errors, and approach to identify TREs and classify their underlying mechanisms are strengths of this study. However, as the research was conducted at 1 hospital, the results may not be generalizable across other settings, including other pediatric hospitals.^6^^,^^7^^,^^14^ Furthermore, as our analysis was based on a retrospective review of records, we may not have considered mechanisms for errors that are not detectable from records and CPOE review. For example, we were not able to consider whether some errors were due to the device being used and “screen real estate” available to view a patient’s record. Lastly, it should be noted that identifying which errors are technology-related can be a difficult process requiring detailed review of records. We conducted inter-rater reliability testing between our 2 reviewers to ensure consistency in identifying a TRE and assigning a mechanism category to the TRE. Our detailed methodology for identifying TREs is available publicly^25^ and those looking to apply this methodology should also ensure consistency in identification and classification of TREs.

Our results provide important insights into where the CPOE may be facilitating prescribing errors, and we have demonstrated that the rates of TREs may change little over time, emphasizing the need for regular CPOE monitoring of TREs to inform system optimization. Hospitals may do this by applying the TREM classification to errors that are identified through regular quality and safety processes, such as reviews of medication orders by clinical pharmacists. We have already shown significant benefits in understanding TRE mechanisms, with results from this study used to inform several modifications in the CPOE at our study site (eg, changes to drop-down menu options to reduce selection errors). Further, based on this research, we developed brief bulletins, the Health Innovation Series, which provide CPOE optimization tips for CPOE managers and vendors, which can also serve to educate staff of key CPOE issues.^39^

## Conclusion

To our knowledge this is the first study to examine the longitudinal rates of TREs using chart review and the first to do this in a pediatric setting using a representative sample of admissions. The unintended consequences of HIT and TREs in CPOE systems have been recognized for over 2 decades; however there has been little progress to systematically address these issues. Our results show that TREs persist several years after CPOE implementation and demonstrate that understanding the mechanisms of TREs can be used to inform CPOE optimization strategies.

## Supplementary Material

ocae218_Supplementary_Data

## Data Availability

This study used individual patient health data that cannot be shared without ethical approval. This also precludes the sharing of aggregated datasets. Analysis datasets are stored according to the ethical approval and access can only be provided to researchers who have received approval from the ethics committee.
